# Lymph node density in papillary thyroid carcinoma is a prognostic factor after adjusting for pathological stage

**DOI:** 10.18632/oncotarget.25453

**Published:** 2018-06-01

**Authors:** Hidenori Suzuki, Yusuke Koide, Nobuhiro Hanai, Daisuke Nishikawa, Shintaro Beppu, Shinji Mikami, Yasuhisa Hasegawa

**Affiliations:** ^1^ Department of Head and Neck Surgery, Aichi Cancer Center Hospital, Nagoya, Japan; ^2^ Department of Otolaryngology-Head and Neck Surgery, Nara Medical University, Kashihara, Japan

**Keywords:** lymph node density, papillary thyroid carcinoma, eighth edition of the Union for International Cancer Control TNM Classification of Malignant Tumors, survival, multivariate

## Abstract

We investigated the possible association between the lymph node density and survival outcomes in differentiated papillary thyroid carcinoma, and examined whether the lymph node density was a predictor in a multivariate analysis adjusted for the pathological stage in the eighth edition of the Union for International Cancer Control Tumor-Node Metastasis Classification of Malignant Tumors. A total of 543 patients with papillary thyroid carcinoma were enrolled. We performed restaging according to the eighth edition. The lymph node density was the ratio between number of positive lymph nodes and total number of excised lymph nodes. A log-rank test and Cox's proportional hazards model were used for univariate and multivariate analysis with adjustment for the pathological stage in the eighth edition, respectively. In both the univariate and multivariate analyses of 150 patients with pN1bM0, the presence of a lymph node density of ≥ 0.3 with pN1b was significantly associated with shorter disease-specific survival. In both the univariate and multivariate analyses of all 543 patients, a lymph node density of ≥ 0.3 with pN1b were also significantly associated with shorter overall and disease-specific survival. In conclusion, these results suggest that the lymph node density can be used as a predictor for the survival outcomes after adjustment for the pathological stage in the eighth edition.

## INTRODUCTION

Staging based on the tumor-node metastasis (TNM) classification is widely used as a prognostic factor for the survival outcomes, such as overall survival (OS) and disease-specific survival (DSS), in various types of carcinoma, including differentiated papillary thyroid carcinoma (PTC) [1]. The pathological stage (pStage) in the eighth edition of Union for International Cancer Control TNM classification of Malignant Tumors (UICC8th) was expected to be a better prognostic factor for many types of carcinoma, and significant associations were found between the survival outcomes and the stage when restaging based on the UICC8th was performed for oropharyngeal carcinoma and PTC [1–4]. However, it was difficult to predict OS and DSS using the same TNM stage [5, 6].

The lymph node density (LND), which is defined as the ratio of the number of positive lymph nodes to the number of total lymph nodes, has been shown to be associated with the survival outcomes in many types of carcinoma, including head and neck carcinoma [1, 5–9]. In a review and several articles of PTC, the LND was also shown to be a significant prognostic factor for the survival outcomes [1, 7–9]. Recently, Amit *et al*. reported in a significant association between the LND and DSS in PTC based on a multivariate analysis with adjustment for clinicopathological factors, including the pathological T classification and N classification in the UICC8th; however, the analysis was not adjusted for the pathological stage in the UICC8th or the LND [1]. To the best of our knowledge, no studies have investigated whether the LND is a prognostic factor in PTC based on a multivariate analysis with adjustment for the pathological stage in the UICC8th.

In the present study, we investigated the possible association between the LND and the survival outcomes of patients with PTC, and examined whether the LND was a prognostic factor in a multivariate analysis with adjustment for the pathological stage in the UICC8th.

## RESULTS

### Clinicopathological factors and pStage

The clinicopathological factors were shown in Table 1. The association between the pStage in the UICC7th and the pStage in the UICC8th is shown in Table 2. Of the 306 patients with pStage I in the UICC8th, 202, 6, 74, and 24 patients were restaged from pStage I, II, III, and IVA in the UICC7th, respectively. Of the 199 patients with pStage II in the UICC8th, 3, 122, and 74 patient were restaged from pStage II, III, and IVA in the UICC7th, respectively. Twenty-four patients with pStage III and 14 patients with pStage IVB in the UICC8th were restaged from 24 patients with pStage IVA and 14 patients with pStage IVC in the UICC7th, respectively.

**Table 1:** Clinicopathological factors in 543 patients with papillary thyroid carcinoma

Factor			
Gender	Male/Female		143/400
Age	<45/45≥ <55/55≥		126/97/320
Pathological stage (UICC7th)	pStage I	<45 pTAnyNAnyM0	123
		45≥ pT1 N0-X M0	79
	pStage II	<45 pTAnyNAnyM1	3
		45≥ pT2 N0N-X M0	6
	pStage III	45≥ pT3 N0-X M0	58
		45≥ pT1-3 N1a M0	138
	pStage IVA	45≥ pT1-3 N1b M0	97
		45≥ pT4a N0-1 M0	25
	pStage IVB	45≥ pT4b N0-1 M0	0
	pStage IVC	45≥ AnyT AnyN pM1	14
Pathological stage (UICC8th)	pStage I	<55 pTAnyNAnyM0	220
		55≥ pT1-2 N0-X M0	86
	pStage II	<55 pTAnyNAnyM1	3
		55≥pT3 N0-X M0	23
		55≥pT1-3 N1 M0	173
	pStage III	55≥pT4a AnyN M0	24
	pStage IVA	55≥pT4b AnyN M0	0
	pStage IVB	55≥AnyT AnyN pM1	14
Positive surgical margin	Presence/Absence		70/473
Operation	Total thyroidectomy/Others		251/292
Adjuvant RAI	Presence/Absence		59/484
Charlson comorbidity index	0/1≥		272/271
Survival outcome	Cancer-specific death/ Other death/Alive		20/23/500

Abbreviations: pStage: pathological stage; UICC8th: the eighth edition of the Union for International Cancer Control TNM Classification of Malignant Tumors; UICC7th: the seventh edition of the Union for International Cancer Control TNM Classification of Malignant Tumors; RAI: radioactive iodine.

**Table 2:** Association between the pStages in the UICC7th and UICC8th

	UICC 7th				
	pStageI (*n* = 202)	pStageII (*n* = 9)	pStageIII (*n* = 196)	pStageIVA (*n* = 122)	pStageIVC (*n* = 14)
UICC8th					
pStageI (*n* = 306)	202	6	74	24	0
pStageII (*n* = 199)	0	3	122	74	0
pStageIII (*n* = 24)	0	0	0	24	0
pStageIVB (*n* = 14)	0	0	0	0	14

Abbreviations: pStage: pathological stage; UICC8th: the eighth edition of the Union for International Cancer Control TNM Classification of Malignant Tumors; UICC7th: the seventh edition of the Union for International Cancer Control TNM Classification of Malignant Tumors.

### Clinical course

At the end of the study, the mean ± SD follow-up period among the whole population, the 500 surviving patients (92.1%, vs all), the 43 patients who died (7.9%) and the 20 patients (3.7%, vs all) who died due to PTC was 2205 ± 1284, 2234 ± 1298, 1875 ± 1073, 1616 ± 1032 days, respectively. In the study population, the 3-, 5-, 10-years OS rates were 97.5%, 94.6%, and 84.5%, respectively. While the 3-, 5-, 10-years DSS rates were 98.3%, 97.0%, and 93.6%, respectively. The causes of death for 20 patients who died due to PTC were distant failure (*n* = 15), locoreginal relapse (*n* = 4), and pulmonary embolism as a complication (*n* = 1). The causes of death for 23 patients who died due reasons to other than PTC were other malignancy (*n* = 14), unknown (*n* =8), and interstitial pneumonia (*n* =1).

### Survival analysis based on pStage on the UICC7th and the UICC8th in all 543 patients

**Figure 1:**
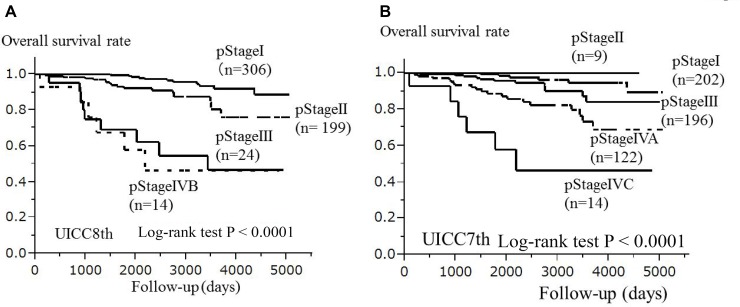
The association between the pathological stage and the overall survival of 543 patients with papillary thyroid carcinoma (Kaplan-Meier method) The pStages in the UICC8th (*p <* 0.0001) (**A**) and UICC7th (*p <* 0.0001) (**B**) were significantly associated with overall survival after the Bonferroni's correction (log-rank test). Abbreviations: pStage: pathological stage; UICC8th: eighth edition of the Union for International Cancer Control TNM Classification of Malignant Tumors; UICC7th: seventh edition of the Union for International Cancer Control TNM Classification of Malignant Tumors.

In the univariate analysis of all 543 paients, both the pStage in the UICC7th among the 5 staging groups (pStageI, II, III, IVA, IVC) (*p <* 0.0001) and pStage in the UICC8th among the 4 staging groups (pStageI, II, III, IVB) (*p <* 0.0001) were significantly associated with OS after the Bonferroni's correction. The Kaplan-Meier curves from the univariate analyses of OS for the pStage in the UICC7th and the pStage in the UICC8th are shown in Figure 1. In the multivariate analysis adjusted for the pStage in the UICC7th and the pStage in the UICC8th (Table 3), the pStage in the UICC8th was significantly associated with OS after the Bonferroni's correction. (per pStage: HR 2.22, 95% CI 1.38–3.70, *p* = 0.0008)

**Table 3:** The multivariate analysis for adjustment with pStages in the UICC7th and UICC8th

Factor	Overall survival		
	Hazards ratio	95% Confidence interval	*P*-value
pStage on UICC8th	2.22	1.38–3.70	0.0008
pStage on UICC7th	1.27	0.83–1.95	0.26

Abbreviations: pStage: pathological stage; UICC8th: the eighth edition of the Union for International Cancer Control TNM Classification of Malignant Tumors; UICC7th: the seventh edition of the Union for International Cancer Control TNM Classification of Malignant Tumors.

### Survival analysis based on LND in 150 patients with pN1bM0

**Figure 2:**
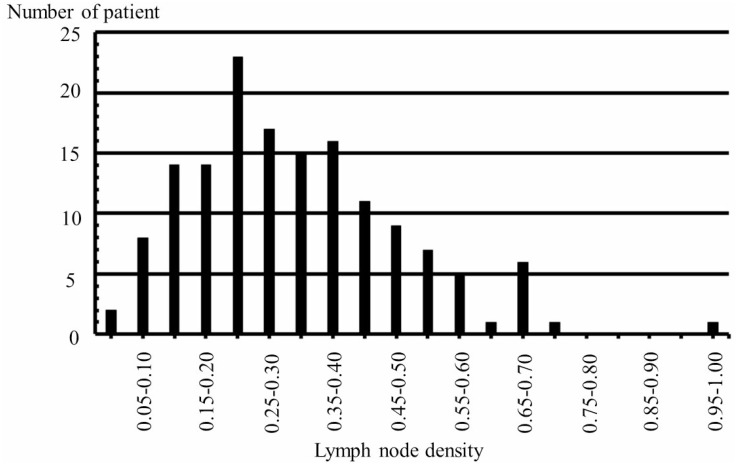
Association between the lymph node density and number of patients with pN1bM0 in 150 papillary thyroid carcinoma patients

**Figure 3:**
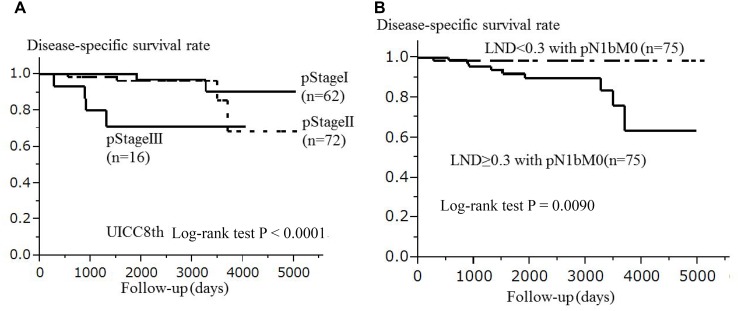
In 150 papillary thyroid carcinoma patients with pN1bM0 (Kaplan-Meier method), the pStage in the UICC8th (*p* < 0.0001) (**A**) was significantly associated with disease-specific survival after the Bonferroni's correction, and the lymph node density (*p* = 0.0090) (**B**) were significantly associated with disease-specific survival; non-significance with Bonferroni's correction of the significance level *p* < 0.0042. The log-rank test was used for the statistical analysis. Abbreviations: pStage: pathological stage; UICC8th: eighth edition of the Union for International Cancer Control TNM Classification of Malignant Tumors; LND: lymph node density.

In 150 patients with pN1bM0, the pStage in the UICC8th (I/II/III) was significantly associated with DSS (*p <* 0.0001) after Bonferroni's correction. The median and mean ± SD LND of the 150 patients with pN1bM0 was 0.3 and 0.32 ± 0.17, respectively (Figure 2). We divided the patients into 2 groups based on the median LND (0.3). Among the pN1bM0 patients, LND ≥ 0.3 (*n* =75) were significantly associated with a shorter DSS in comparison to LND < 0.3 (*n* = 75) (*p* = 0.0090); non-significance with Bonferroni's correction of the significance level *p <* 0.0042. The Kaplan-Meier curves from the univariate OS analyses of both pStage in the UICC8th and LND (LND ≥ 0.3 / LND < 0.3) for the 150 patients with pN1bM0 are shown in Figure 3. In the multivariate analysis adjusted for pStage in the UICC8th and LND in the 150 patients with pN1bM0, pStage in the UICC8th (per pStage: HR 4.18, 95% CI 1.61–11.8, *p* = 0.0033) and the presence of LND ≥ 0.3 with pN1bM0 (HR 17.5, 95% CI 2.58–138.4, *p* = 0.0013) was significantly associated with the DSS after the Bonferroni's correction. A multivariate analysis of the DSS in the 150 patients with pN1bM0 is shown in Table 4.

**Table 4:** The multivariate disease-specific survival analysis for adjustment with the pStage in the UICC8th (I/II/III) and LND (LND ≥ 0.3 with pN1bM0/LND < 0.3 with pN1bM0) in pN1bM0

Factor	Disease-specific survival		
	HR	95% CI	*P*-value
pStage in the UICC8th (I/II/III)	4.18	>1.61–11.8	0.0033
LND (LND ≥ 0.3 with pN1bM0/LND < 0.3with pN1bM0)	17.5	2.58–138.4	0.0013

Abbreviations: pStage: pathological stage; HR: Hazards ratio; 95% CI: 95% confidence interval; UICC8th: the eighth edition of the Union for International Cancer Control TNM Classification of Malignant Tumors; LND: lymph node density.

### Univariate survival analysis based on LND in all 543 patients

**Figure 4:**
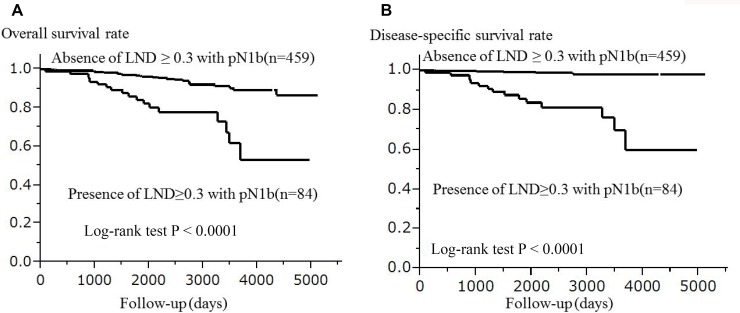
The association between LND and the survival of 543 patients with papillary thyroid carcinoma (Kaplan-Meier method) LND ≥ 0.3 was found to be associated with a significantly lower overall survival (*p* < 0.0001) (**A**) and disease-specific survival (*p* < 0.0001) (**B**) by log-rank test after the Bonferroni's correction. Abbreviation: LND: lymph node density.

Among the 15 patients with pN1bM1, 9 patients had LND ≥ 0.3 with pN1b. In total, 84 patients had LND ≥ 0.3 with pN1b, this included 75 patients with pN1bM0 and 9 patients with pN1bM1. In all 543 patients, the presence of LND ≥ 0.3 with pN1b (*n* = 84) was significantly associated with shorter OS (*p <* 0.0001) and DSS (*p <* 0.0001) in comparison to the absence of LND ≥ 0.3 with pN1b (*n* = 459) after the Bonferroni's correction. The Kaplan-Meier curves from both OS and DSS of the presence/absence of LND ≥ 0.3 with pN1b are shown in Figure 4.

### Multivariate survival analysis in all 543 patients

The results of the multivariate analyses of both OS and DSS in all 543 patients are shown in Table 5. In the multivariate analysis with adjustments for pStage in the UICC8th and LND ≥ 0.3 with pN1b, pStage in the UICC8th was significantly associated with both OS (per pStage: HR 2.40, 95% CI 1.76–3.24, *p <* 0.0001) and DSS (per pStage: HR 2.53, 95% CI 1.67–3.85, *p <*0.0001) after the Bonferroni's correction, the presence of LND ≥ 0.3 with pN1b was significantly associated with the OS (HR 2.50, 95% CI 1.27–4.82, *p* = 0.0086); non-significance with the Bonferroni's correction of the significance level *p <* 0.0042, and the presence of LND ≥ 0.3 with pN1b was significantly associated with both the DSS (HR 9.78, 95% CI 3.49–31.7, *p <* 0.0001) after the Bonferroni's correction. In the multivariate analysis after adjusting for the pStage in the UICC8th, LND ≥ 0.3 with pN1b, gender, positive surgical margin, thyroidectomy, adjuvant RAI, and CCI, pStage in the UICC8th (per pStage: HR 2.33, 95% CI 1.66–3.24, *p <* 0.0001) was significantly associated with shorter OS after the Bonferroni correction, the presence of LND ≥ 0.3 with pN1b (HR 2.22, 95% CI 1.08–4.52, *p* = 0.0305) was significantly associated with shorter OS; non-significance with the Bonferroni's correction of the significance level *p <* 0.0042, and the pStage in the UICC8th (per pStage: HR 2.17, 95% CI 1.38–3.46, *p* = 0.0009), presence of LND ≥ 0.3 with pN1b (HR 6.04, 95% CI 2.08–20.6, *p* = 0.0007), male gender (HR 3.55, 95% CI 1.27–10.4, *p* = 0.00154) were associated with shorter DSS significantly after the Bonferroni's correction.

**Table 5:** The multivariate survival analysis in all patients (*n* = 543)

Factor	Overall survival			Disease-specific survival		
	HR	95% CI	*P*-value	HR	95% CI	*P*-value
Model 1						
pStage in the UICC8th (I/II/III/IVB)	2.40	1.76–3.24	<0.0001	2.53	1.67–3.85	<0.0001
LND ≥ 0.3 with pN1b (Presence/Absence)	2.50	1.27–4.82	0.0086	9.78	3.49–31.7	<0.0001
Model 2						
pStage in the UICC8th (I/II/III/IVB)	2.33	1.66–3.24	<0.0001	2.17	1.38–3.46	0.0009
LND ≥ 0.3 with pN1b (Presence/Absence)	2.22	1.08–4.52	0.0305	6.04	2.08–20.6	0.0007
Gender (Male/Female)	1.42	0.71–2.76	0.3191	3.55	1.27–10.4	0.00154
Positive surgical margin (Presence/Absence)	2.02	0.89–4.14	0.0902	2.76	0.91–7.54	0.0708
Thyroidectomy (Total /Others)	1.32	0.64–2.76	0.4491	2.39	0.64–11.5	0.2041
Adjuvant RAI (Presence/Absence)	0.59	0.19–1.55	0.3010	0.74	0.19–2.39	0.6308
CCI (CCI ≥ 1/CCI = 0)	0.95	0.51–1.77	0.8621	0.61	0.23–1.54	0.2899

Abbreviations: HR: Hazards ratio; 95% CI: 95% confidence interval; UICC8th: the eighth edition of the Union for International Cancer Control TNM Classification of Malignant Tumors; LND: lymph node density; RAI: radioactive iodine; CCI: Charlson comorbidity index.

## DISCUSSION

In the present study, we showed for the first time in both univariate and multivariate analyses adjusted for the pStage in the UICC8th that PTC patients of LND ≥ 0.3 with pN1bM0 had significantly shorter DSS, and that patients with LND ≥ 0.3 with pN1b had significantly shorter OS and DSS.

Restaging of the pStage on UICC 8th from the pStage of the seventh edition according to the eighth edition has been reported to be superior for predicting survival in various types of carcinoma, including PTC [2–4]. Pontius *et al*. reported in PTC that the differences (UICC7th vs UICC8th) were age for cut point (45 vs 55), extrathyroidal extension (minimal vs gross), and N stage (N1a and N1b vs single N1), and that the UICC8th model showed a better fit to the data in comparison to the UICC7th model [2]. The findings of the present study showed that the pStage in the UICC8th was significantly associated with OS in a multivariate analysis adjusted for the pStage in the UICC7th, which is in agreement with the findings of previous studies [2–4].

The LND, which is influenced by nodal staging, surgical, and pathological factors, has been shown to be a prognostic factor in many types of carcinoma, regardless of the types of neck dissection that are used [5]. Because the LND was reported to be affected by the number of lymph nodes retrieved based on the extent of surgery [7], we analyzed about patients with N1b rather than N1, despite on the UICC8th. LND value may change depending on the criteria to submit lymph nodes for a pathological examination. We also reported that there was a significant association between LND and the survival outcomes including the OS and DSS in hypopharyngeal and oral squamous cell carcinoma [5, 6]. Lee *et al*. reported that a higher LND was significantly associated with shorter survival in PTC with pN1b [8]. Moreover, a recent review for nine articles to investigate the prognostic value of the LND in PTC showed a significant association between the LND and the survival outcomes, and the authors noted that a lymph node ratio of ≥ 0.3 was associated with an increased risk of recurrence [2–4]. The findings of the present study revealed significant associations between the LND and survival outcomes, and are in good agreement with the results of previous studies [5–8]. The cut-off values of continuous variable in a scientific study as systemic review and meta-analysis have been determined by the lowest *p*-value, receiver-operating characteristic, and a median value [14], so we selected the median value of LND (0.3) as the lucid determination in the present study.

Amit *et al*. reported that a higher LND was significant associated with shorter OS and DSS in univariate and multivariate analyses in 2542 patients with PTC, and that the multivariate analysis was adjusted for clinicopathological factors, including the pathological T and N classification in the UICC8th [1]. To the best of our knowledge, no studies have reported the results of a multivariate analysis adjusted for the LND and pStage in the UICC8th. Thus, we considered that there was a need for such an analysis. In the present study, in which we performed a multivariate analysis adjusted for the pStage in the UICC8th, we found a significant association between the LND and the survival outcomes of patients with in PTC. Because the stage based on both the T and N classification is comprehensive, we considered that a multivariate analysis adjusted for the pathological stage would be superior to the analyses made in the previous study by Amit *et al*, in which a multivariate analysis was performed with adjustment for the pathological T and N classification [1]. Based on the results of the present study, we have two plans to add adjuvant RAI and to monitor closely during follow up for patients with higher LND as described previously [7].

We believe that there was no possibility that the LND is confounded with any risk factors another than the clinical stage in UICC8th of PTC.

The present study was associated with some limitations. Specifically, it was retrospective in nature and the study population was relatively small. A future prospective study with a large cohort and multi-institutional setting would yield more accurate and useful results. Because all lymph nodes were examined in a single representative cross section, as described previously [6], the present study could not detect the micro metastases of lymph nodes, which are well known to occur in cases of PTC. Because a lateral neck dissection generally is performed for the patients with lymph node metastasis detected at the lateral neck before surgery, we considered that a preoperative diagnosis might be a potential source of bias. Because we analyzed not only patients with pN1bM0 but also those with pN1b, we considered that there was some risk of an increased α-error in the present study.

In conclusion, the present study demonstrated, in both univariate and multivariate analysis adjusted for the pStage in the UICC8th, that the LND is significantly associated with shorter OS and DSS in patients with PTC. The LND can therefore be considered to be a prognostic factor that can predict the survival outcomes in patients with PTC.

## MATERIALS AND METHODS

### Study design and patients

Between 2003 January and 2016 August, 601 patients without a history of thyroid surgery underwent radical surgery for PTC at Aichi Cancer Center Hospital. We excluded 58 of these 601 patients who had the following pathological diagnoses: anaplastic carcinoma (*n* = 19), medullary carcinoma (*n* = 15), follicular carcinoma (*n* = 13), poorly differentiated carcinoma (*n* = 7), carcinoma showing thymus-like differentiation (*n* = 3), squamous cell carcinoma (*n* = 1). Thus, 543 patients, who were pathologically diagnosed with PTC, were enrolled in this study. This study was approved by our institutional review board, and informed consent for the treatments and examinations was obtained from all of the patients.

### Clinical staging and surgery

Clinical staging was determined from a routine physical examination, laryngoscopy, ultrasonography, enhanced cervical computed tomography, cytology and 18F-FDG-PET/CT as needed. The Charlson comorbidity index, which was a weighted index, was calculated based on 19 comorbid conditions. The TNM classification was determined based on the International Union Against Cancer classification. The 543 patients were classified into the total thyroidectomy group (*n* = 251), non-total thyroidectomy group (*n* = 292) according to the extent to their operation. For neck dissection, we performed *en bloc* dissection, as described by the Japan Neck Dissection Study Group [10]. We performed both lateral neck dissection and central neck dissection as preoperative staging for patients who were diagnosed with cN1b or intraoperative staging for patients who were diagnosed with sN1b based on the pathological diagnosis after palpation in the operation. We performed central neck dissection for patients who were diagnosed with carcinoma based on preoperative cytology or an intraoperative pathological examination.

### Follow-up after surgery

After surgery, the patients were followed-up at our outpatient clinic by ultrasonography and thyroglobulin measurements with a surveillance schedule of approximately once every 6–12 months, and enhanced computed tomography or 18F-FDG-PET/CT as needed. Thyroid stimulating hormone suppression was performed after total thyroidectomy, and we made an effort to perform salvage surgery for locoregional recurrence when possible. Adjuvant radioactive iodine (RAI) was indicated for patients with distant metastasis from 2003 to 2009, and was recommended for high-risk patients with conditions such as distant metastasis and severe extrathyroid extension from 2010, based on the established guideline of the Japanese Society of Thyroid Surgeons and the Japanese Association of Endocrine Surgeons, as described previously [11].

### Pathological staging

Neck dissection samples were separated according to the cervical region, and then the total number of lymph nodes was recorded. We submitted all lymph nodes obtained during neck dissection for a pathological examination. Pathological examinations of the lymph nodes were performed in a single representative cross section. All of the other pathological examination methods have been published previously [6]. The pathological diagnosis (pathological T and N classification, pStage, surgical margin) was made by two experienced pathologists, who completed each report. We determined the pathological TNM classification based on the pathological report according to the UICC7th. We redetermined the pStage based on the UICC8th using both the pathological report and the record of the intraoperative findings, as described by the eighth edition of the cancer staging manual of the American Joint Committee on Cancer and the UICC8th [12, 13].

### LND

For the 150 patients (27.6%) with pN1bM0, a total of 6920 lymph nodes were evaluated; 2068 (29.9%) were found to be pathologically positive. In these 150 patients, the mean ± SD numbers of excised lymph nodes and positive lymph nodes were 46.1 ± 29.3 and 13.8 ± 10.5, respectively. In 17 patients with M1, 15 patients with cN1bM1 underwent lateral neck dissection; 2 patients with cN0-1aM1 did not undergo lateral neck dissection. For the 15 patients with pN1bM1, who underwent lateral neck dissection, a total of 748 lymph nodes were evaluated, and 274 (36.6%) were found to be pathologically positive. In these 15 patients, the mean ± SD numbers of excised lymph nodes and positive lymph nodes were 49.9 ± 27.7 and 18.3 ± 12.2, respectively. The LND was calculated using the following formula:

LND = the number of positive lymph nodes/the total number of excised lymph nodes.

### Statistical analysis

Statistical analyses were performed using the JMP software package (version 9; SAS; Cary, NC, USA). Survival time as the period from surgery to survival endpoint was calculated by the Kaplan-Meier method. The survival endpoints were death for OS, and death from PTC for DSS. A univariate survival analysis was carried out using a log-rank test. A multivariate analysis was performed using Cox's hazards model; both the hazards ratio (HR) and 95% confidence interval (95% CI) were assessed. In all 543 patients, univariate and multivariate analyses of OS according to the pStage in the UICC7th (I/II/III/IVA/IVC) and UICC8th (I/II/III/IVB) were performed. One hundred fifty patients with pN1bM0 were divided into 2 groups based on the median LND (LND ≥ 0.3: *n* = 75; LND < 0.3: *n* = 75). Univariate and multivariate analyses of DSS according to the pStage in the UICC8th (I/II/III) and LND (LND ≥ 0.3 with pN1bM0/LND < 0.3 with pN1bM0) were performed.

Because 9 of 15 patients with pN1bM1 had the presence of LND ≥ 0.3 with pN1b, all 543 patients were divided into 2 groups according to the presence of LND ≥ 0.3 with pN1b (*n* = 84) or the absence of LND ≥ 0.3 with pN1b (*n* = 459). We performed a univariate survival analysis compare both the OS and DSS according to the presence or absence of LND ≥ 0.3 with pN1b in all 543 patients. We assessed two multivariate analysis models for both OS and DSS in all patients. One model was adjusted for the pStage in the UICC8th (I/II/III/IVB) and LND ≥ 0.3 with pN1b (presence/absence); the other model was adjusted with pStage in the UICC8th (I/II/III/IVB), LND ≥ 0.3 with pN1b (presence/absence), gender (male/female), CCI (CCI ≥ 1/ CCI = 0), positive surgical margin (presence/absence), thyroidectomy (total/others), adjuvant RAI (presence/absence). *P* values of < 0.05 were considered to indicate statistical significance. Given the risk of α- error from many statistical analyses, we additionally adjusted Bonferroni's correction for the *P* values as reported [15]. *P* values of < 0.0042 were considered to indicate statistical significance after Bonferroni's correction, as 12 individual tests were performed (0.05/12).
